# Caregiver and community perceptions and experiences participating in an infant malaria prevention trial of PfSPZ Vaccine administered by direct venous inoculation: a qualitative study in Siaya County, western Kenya

**DOI:** 10.1186/s12936-020-03293-7

**Published:** 2020-06-24

**Authors:** Florence Achieng, Joseph G. Rosen, Reuben Yego Cherop, Simon Kariuki, Stephen L. Hoffman, Robert Seder, Martina Oneko, Laura C. Steinhardt

**Affiliations:** 1 Centre for Global Health Research, Kenya Medical Research Institute, Kisumu, Kenya; 2 Department of International Health, Johns Hopkins Bloomberg School of Public Health, Baltimore, MD USA; 3 Sanaria, Inc., Rockville, MD USA; 4 Vaccine Research Center, National Institute of Allergy and Infectious Diseases, National Institutes of Health, Bethesda, MD USA; 5 Malaria Branch, Division of Parasitic Diseases and Malaria, Center for Global Health, Centers for Disease Control and Prevention, Atlanta, GA USA

**Keywords:** Direct venous inoculation, PfSPZ Vaccine, Malaria, Caregivers, Perceptions, Qualitative research, Kenya, Infants

## Abstract

**Background:**

Despite available control strategies, malaria morbidity and mortality, especially in infants and young children in sub-Saharan Africa, remain intractable. Malaria vaccination could substantially reduce malaria episodes and deaths. One vaccine candidate is the whole sporozoite PfSPZ Vaccine, consisting of irradiated cryopreserved sporozoites administered by direct venous inoculation (DVI). DVI may be less acceptable than more familiar administration routes, particularly intramuscular. As part of a PfSPZ Vaccine trial among infants in western Kenya, a qualitative study was conducted to explore caregiver and community perceptions of the malaria vaccine trial, including the unique DVI administration procedure.

**Methods:**

Twelve focus groups and 28 in-depth interviews explored perceptions of the DVI procedure in infants, factors influencing trial acceptability, and barriers to sustained trial participation. Purposively sampled participants included mothers of enrolled children, fathers and mothers who withdrew their children from the trial, village elders, and study clinicians from two trial enrollment sites. An iterative, multi-stage analytic approach, adapted from the Framework Method, was used to synthesize and interpret textual data.

**Results:**

Desires to prevent malaria and participation incentives (e.g., free consultations and medication) motivated caregivers to enroll their children in the trial. However, numerous factors also demotivated trial participation. Family members’ (i.e., fathers') objections to required blood draws were cited most frequently as drivers of early trial withdrawal, in many cases prior to receiving any vaccine. Among mothers whose children received PfSPZ Vaccine (or placebo), many spoke favourably of DVI administration, describing improved tolerability relative to intramuscularly administered immunizations. Other trial-related challenges cited by caregivers included negative interactions with study clinicians and perceived delays in administering trial procedures.

**Conclusions:**

Despite high acceptance of DVI among caregivers whose children received PfSPZ Vaccine (or placebo), objections to trial procedures from other non-sensitized household and family members prompted early trial withdrawal and inhibited successful completion of trial procedures for some infants. Implications for future trials include targeting heads of household during sensitization and recruitment activities, as well as equipping trial staff to effectively respond to participant and community concerns regarding trial procedures.

## Background

Malaria remains a leading killer of infants and children under 5 years of age worldwide. In 2017, malaria was responsible for an estimated 266,000 deaths in children less than 5 years-old, the majority in sub-Saharan Africa [1]. Global reductions in malaria morbidity and mortality have been attributed to successful scale-up of malaria control interventions, including long-lasting insecticide-treated nets (LLINs) [2], indoor residual spraying (IRS) [3], effective case management with artemisinin-based combination therapy (ACT) [4], and seasonal malaria chemoprevention in children [5–7]. However, inadequate access to and sub-optimal community uptake of these key strategies, vector resistance to insecticides used in LLINs and IRS, and parasite resistance to ACT in parts of southeast Asia threaten continued progress in reducing the malaria burden [8]. New tools for malaria prevention and control are urgently needed.

Malaria vaccines present new opportunities to reduce malaria transmission. One of the most advanced malaria vaccines is the whole sporozoite PfSPZ Vaccine, which consists of aseptic, purified, cryopreserved, radiation-attenuated whole *Plasmodium falciparum* sporozoites (SPZ) [9]. Earlier studies found that intradermal or subcutaneous administration of the vaccine conferred minimal immunity [10], but higher doses administered intravenously resulted in sterile protection in a small sample of malaria-naive volunteers in the USA [11]; subsequent studies have used this method of direct venous inoculation (DVI) in volunteers in endemic settings [12, 13]. PfSPZ Vaccine has demonstrated dose-dependent, moderate- to high-level protection for healthy adults in several clinical trials in Mali [12] and the USA [10, 11]; however, efficacy in children and infants was unknown at the time of the aforementioned trials.

PfSPZ Vaccine’s DVI administration method raises questions about how its unusual administration route may influence vaccine perceptions, compared to more traditional intramuscular (IM) immunizations, and what additional sensitization or communication efforts would be needed to optimize acceptability and uptake. Parental vaccine hesitancy can result in delays or outright refusal of infant immunization. A recent systematic review of the RTS,S  malaria vaccine trials in sub-Saharan Africa highlighted the contribution of inadequate communications for setting expectations and community mistrust of health institutions delivering immunizations to RTS,S receptivity [14]. Another study in the Peruvian Amazon demonstrated how adults’ preferences for different vaccine administration routes (i.e., oral over injection) could influence acceptance of prospective malaria vaccines for infants [15]. Previous research has also identified multi-level factors influencing community acceptability of and engagement with candidate malaria vaccine trials in high-prevalence settings. These span personal considerations (e.g., perceived efficacy of malaria vaccines among caregivers, perceived threat of malaria) [16–18], product compositional attributes (e.g., route of administration, durability, side effects) [16, 18–20], financial considerations for trial participation (e.g., costs incurred travelling to health facilities) [21], and implementation contexts (e.g., provider responsiveness to patient/caregiver concerns during clinical encounters, expediency of vaccination procedure(s) in healthcare settings) [17, 19, 20]. Much of the available evidence, however, is formative and, therefore, has not documented community perspectives or experiences with specific malaria vaccines in trial or implementation contexts [15–21]. In light of PfSPZ Vaccine’s unique administration method, additional research is needed to unearth factors that may enable or inhibit uptake and completion of PfSPZ Vaccine series, particularly when the vaccine is introduced in settings where caregivers and other household members with decision-making authority in infant healthcare-seeking may be unfamiliar with DVI administration of vaccines.

This paper presents findings from a qualitative study exploring caregiver, community member, and study clinician perceptions and experiences with a recently-completed Phase II PfSPZ Vaccine trial among infants in Siaya County, western Kenya. Qualitative methods were used to document perceptions of the DVI procedure in infants, as well as other situational and contextual factors influencing trial participation.

## Methods

### Study site

This qualitative study was nested within a vaccine trial conducted in Siaya County, located in Nyanza Province in the lake endemic zone of western Kenya, approximately 400 km west of Nairobi. The lake region shoulders a disproportionately high burden of malaria in the country, with an estimated 27% parasite prevalence among children 14 years and younger in 2015, compared to 8% nationally [22]. Malaria transmission in the area is perennial, peaking in May–July and October–November, coinciding with the onset of long and short rains, respectively. Most malaria infections are due to *P. falciparum*. Residents are predominantly of the Luo ethnic group, whose common livelihoods include agriculture, fishing, and microenterprises.

A Phase III RTS,S Vaccine trial [23] and other vaccine safety trials [24] have previously been conducted in Siaya County. Most recently, Siaya County Referral Hospital was the site of a Phase II trial (within which this study was nested) from July 2016 to August 2018 that assessed the tolerability, safety, immunogenicity, and efficacy of PfSPZ Vaccine in infants and children aged 5 months to 9 years (clinicaltrials.gov ID: NCT02687373). After an initial dose-escalation, age de-escalation study, the main study focused on safety, tolerability, and efficacy in a cohort of infants aged 5–12 months, with the primary efficacy objective to determine whether PfSPZ Vaccine provided high-level protection [> 60%] against *P. falciparum* infection in the 6 months following the last of three vaccine doses. Healthy infants living within a 10-km radius of Siaya County Referral Hospital or Wagai Dispensary who had not participated in a previous malaria vaccine trial were eligible for participation. Infants underwent screening and, if eligible, were randomized to one of three vaccine dose groups or placebo, which consisted of normal saline. Vaccination consisted of three injections (vaccine or placebo) administered by DVI at two-month intervals. The vaccine consisted of 0.5 mL of PfSPZ Vaccine or normal saline placebo administered by DVI through a 25-gauge needle, typically into a vein in the infant’s antecubital fossa or back of the hand. This procedure typically required one study staff member to hold the child still and sometimes assist in visualizing the infant’s veins by using a vein viewer, while a second study staff member located the vein and administered the injection. The first dose was successfully administered to 336 infants. Community sensitization was conducted in the study communities prior to recruitment and enrollment in the vaccine trial. Chiefs, assistant chiefs, opinion leaders, and community members were informed about the malaria vaccine trial during community meetings to ensure that the trial information was properly disseminated to community residents.

### Sampling procedure

Three participant subgroups were identified for purposive recruitment into the qualitative study’s sampling frame: (1) primary caregivers (i.e., mothers and fathers) of eligible infants, (2) community members (i.e., village elders), and (3) study clinicians. Primary caregivers recruited to participate in the qualitative study included mothers of children enrolled in the vaccine trial as well as mothers and fathers of children who were withdrawn from the trial, both before and during vaccination. Recruiting caregivers whose children remained in the trial and caregivers who withdrew consent was intended to capture diverse perspectives and experiences at various stages of the trial, from initial sensitization and recruitment to vaccine administration. Village elders were also interviewed to provide perspectives as respected opinion-leaders and community representatives capable of influencing community acceptance of the trial and subsequent trial participation. Lastly, study clinicians involved in the parent PfSPZ Vaccine trial were recruited from both Siaya and Wagai sites to triangulate findings from caregiver and community groups as well as to account for potential differences in trial experiences and perspectives resulting from distinct implementation environments and community receptivity potentially impacting participant engagement.

### Data collection

Qualitative data was collected using semi-structured in-depth interviews (IDIs) and focus group discussions (FGDs). All data were collected after participating infants had received all three vaccination doses or after the parent/guardian had withdrawn the infant from the trial. Following an explanation of the qualitative study objectives and procedures, participants who provided written informed consent were invited to participate in an IDI or FGD. Mothers meeting eligibility criteria for FGDs who expressed preference for an individual interview were sampled for IDIs.

Topics addressed across IDIs and FGDs with mothers, fathers, and village elders included initial motivations to enroll children in the PfSPZ Vaccine trial (including perceived susceptibility to malaria and experiences with previous clinical trials), perceptions of the DVI method, experiences with non-vaccination trial procedures and interactions with trial staff, and barriers to trial enrollment and sustained participation. IDIs with study clinicians included questions on the feasibility of the DVI procedure. FGDs were conducted only with mothers of infants enrolled in the trial or those who were withdrawn, as only these participants (i.e., primary caregivers for trial participants) could feasibly be recruited in large enough numbers for facilitated discussions. To achieve thematic saturation in a heterogenous sample of participants, six participants (three per study community) were purposively sampled across subgroups for IDIs [25].

### Data management and analysis

Except for study clinician interviews (conducted in English), all IDIs and FGDs were conducted in the local language of Dholuo, digitally recorded, transcribed verbatim, and then translated into English. All transcripts were reviewed by four study investigators (FA, JGR, MO, and LCS), including the field supervisor (FA), for clarity and translation quality. This approach allowed investigators to inductively identify patterns in the qualitative data prior to analytic coding.

An iterative, multi-stage analytic approach, adapted from the Framework Method [26], was used to synthesize and interpret textual data. First, themes included in IDI and FGD guides were abstracted and segmented into a hierarchical coding structure consisting of topical (overarching theme), response (sub-theme), and sub-response (phenomenon of sub-theme) codes. Two study investigators (FA and JGR) read each transcript, line-by-line, and drafted analytic memos detailing themes emerging from the data not captured by the deductive textual codes. A four-person team met to review memos and revise codes to include these inductively identified themes. A final codebook consisted of seven topical, 19 response, and 64 sub-response codes.

Two investigators (FA and JGR) used the codebook to develop a data abstraction matrix, which provided a template for summarizing and interpreting findings corresponding to each code within a transcript. Working independently, investigators populated matrices for an initial sample of three transcripts, then reconvened to compare findings, identify similarities and differences in data interpretation, and assess instrument reliability. Once coding differences were reconciled, and matrices were revised to streamline data abstraction, investigators populated matrices for the remaining transcripts in the dataset.

Applying constant comparison [27, 28], salient themes abstracted in the matrices were plotted alongside text descriptions of pertinent participant categories (e.g., participant subgroup, community, age) for each transcript. Once completed for each transcript, a textual grid was generated to facilitate pattern identification between (across-case) and within (within-case) transcripts [29]. To further explore patterns and refine the themes identified, investigators independently drafted synthesis memos for seven dominant themes, articulating interpretations of the theme and presenting abstracted textual data to support interpretation. These memos were circulated among four study investigators to triangulate thematic interpretations and distill study findings.

### Ethical statement

Ethical approval for the study was obtained from the Kenya Medical Research Institute’s Scientific and Ethics Review Unit (Nairobi, Kenya) and the U.S. Centers for Disease Control and Prevention Institutional Review Board (Atlanta, GA, USA). Overall community consent was obtained through meetings with community leaders. Written informed consent was obtained from all participants prior to data collection. Due to high levels of illiteracy in the study communities, participants incapable of providing written informed consent were verbally guided through the study consent procedures and indicated their consent with a thumbprint.

## Results

Overall, 112 caregivers, community members, and clinicians participated in 39 unique data collection events across study communities (Table 1). Six participants per subgroup, with the exception of three study clinicians, completed IDIs (N = 27). A total of twelve FGDs (six per study community) were facilitated for mothers of children enrolled in the trial and those who withdrew. Eighty-five mothers participated in FGDs, with five to nine discussants per group. A socio-demographic profile of participants, disaggregated by subgroup and data collection modality, is presented in Table 2.

**Table 1:** Number of data collection events (N = 39), by participant subgroup and study community

	Mothers in trial		Mothers who withdrew		Fathers who withdrew		Village elders		Study clinicians	
	IDI	FGD	IDI	FGD	IDI	FGD	IDI	FGD	IDI	FGD
Siaya	3	3	3	3	3	0	3	0	2	0
Wagai	3	3	3	3	3	0	3	0	1	0
Total	6	6	6	6	6	0	6	0	3	0

*IDI =* in-depth interview, *FGD* = focus group discussion

**Table 2:** Socio-demographic profile of study participants (N = 112), by participant subgroup

	Mothers in trial	Mothers who withdrew	Fathers who withdrew	Village elders	Study clinicians
Data collection modality					
Focus groups	49	36	–	–	–
In-depth interviews	6	6	6	6	3
Study community					
Siaya	28	20	3	3	2
Wagai	27	22	3	3	1
Age, in years (mean, range)	30.3 (20–42)	30.8 (20–47)	32.8 (27-46)	39.2 (34–45)	35.3 (33–38)
Age group, in years					
20–24	9	7	–	–	–
25–29	14	10	2	–	–
30–34	20	13	3	1	1
35–49	12	12	1	5	2
Sex					
Male	–	–	6	3	2
Female	55	42	–	3	1
No. of children (mean, range)	2.8 (1–5)	2.5 (1–6)	2.5 (1–4)	4.0 (2–6)	2.3 (2, 3)
Occupation					
Agriculture/farming	32	23	1	4	–
Business	22	15	1	1	–
Carpenter/clerk/tailor	1	3	1	–	–
Mechanic/transportation	–	–	3	–	–
Priest	–	–	–	1	–
Teacher	–	1	–	–	–

Salient, cross-cutting themes identified in content analysis are presented according to the following overarching domains: (1) motivations and barriers to trial enrollment, (2) DVI perceptions and experiences, and (3) experiences with non-inoculation trial procedures. These themes correspond to the common trajectories of participant/caregiver experiences in clinical trials, from recruitment and enrollment to participation and completion.

### Motivations and barriers to trial enrollment

#### Malaria prevention

Mothers of children, both those still enrolled in the trial and those who were withdrawn, cited desires to prevent malaria in their children as a chief motivator of their participation. Participants across study sites frequently alluded to the severity, frequency and persistence of malaria, colloquially referred to as *mudhusi* (‘the attacker’) or *nyaldiema* (‘abrupt killer’). The perceived threat of malaria motivated most mothers to enroll their children in the trial, even if the experimental vaccine would not confer complete protection from malaria:

*“I was happy to get involved in this malaria vaccine [trial] because a child who has been vaccinated is not the same as that child who has not been vaccinated. This is because [his] chances of getting malaria are reduced. Even if the child can be affected, he wouldn’t be like the rest.”* (Mother in trial, FGD, Wagai)

Mothers additionally acknowledged the financial strain a child suffering from malaria placed on the household. The prevalence of malaria in the study communities, coupled with the risk of recurrence when not treated appropriately, meant children infected with malaria could experience multiple episodes of illness.

*“If you have [a] malaria patient in the family…life becomes expensive because of the illness every now and then…It’s good if it can be prevented.”* (Mother in trial, FGD, Wagai)

A malaria vaccine thus presented overlapping incentives of preventing childhood illness and safeguarding household finances, motivating caregivers to enroll their children in the trial.

#### Participation incentives

In addition to the potential benefits of having their children receive PfSPZ Vaccine, mothers discussed other healthcare-related and financial dimensions of the trial as factors motivating participation. Irrespective of their child’s current trial enrollment status, mothers described healthcare subsidies (e.g., free consultation and medication, reimbursement of transport costs to and from the study clinics), home visits by study staff and providers, as well as streamlined access to assigned health providers as supplementary incentives for enrolling their children in the trial. Opportunities to bypass bureaucracy and queues in public sector health facilities were emphasized by mothers who otherwise could not afford medical care or access timely medical services when children fell ill.

*“We were told that if the child is sick, you can make a phone call wherever you are…Even if you are far or even if it is midnight, you will be reached, and the child will get help.”* (Mother in trial, FGD, Wagai)

#### Blood draw perceptions

Participants also described a number of barriers demotivating trial enrollment, with the required blood draws emerging as the most salient barrier across participant subgroups and study communities. Participants described their own or other household members’ objections to the blood draws, perceived as medically unnecessary and harmful procedures for young, healthy children. Per the trial protocol, the total volume of blood required for study purposes over approximately 17 months was 29.1 ml, including 4 venous blood draws of 5–5.5 ml each; 1 optional venous blood draw of 2 ml during the first episode of malaria after the third vaccination; 17 capillary blood draws of 0.45-0.5 ml each, and during febrile episodes another capillary blood draw of 0.5 ml (for clinical care and study purposes). Blood draw objections were the most commonly stated reasons for withdrawing from the trial prior to any vaccine administration. These objections were related to broader concerns over the perceived invasiveness of the procedure, with both male and female caregivers expressing concern that blood draws were intended for the very sick, rather than for healthy children, and the procedure’s potential to harm young children, especially those with low birth weight or anaemia.

*“They [trial implementers] should understand that these are small children; therefore, you cannot take blood as if they are in a slaughterhouse.”* (Father who withdrew, IDI, Wagai)

*“When my husband heard about the amount of blood that was going to be removed, he said that it was too much and told me not to go back with the child there [clinic]… They [husband and mother*-*in*-*law] were saying that the child was still very young, and the child does not have enough blood.”* (Mother who withdrew, FGD, Wagai)

#### Rumours surrounding blood theft and selling

A majority of mothers explained that objections to the blood draws stemmed from other household members, particularly fathers and mothers-in-law, many of whom were influenced by rumours circulating in the study communities of malicious intentions of the trial administrators. Not all these household members had participated in the trial’s sensitization activities.

*“He [child’s father] told me to pack and go because I was selling his child’s blood.”* (Mother who withdrew, FGD, Siaya)

Participants indicated these rumours, which largely centered around the misuse of blood (i.e., stealing or sharing) collected for vaccination trials, were directed at visible public health institutions recruiting for clinical research trials in these communities. While participants universally acknowledged the presence of these rumours in their communities, these perceptions were insufficient to motivate consent withdrawal when household support for trial participation, particularly from husbands, was present:

“*My husband supported me even after getting some nasty information that my child will die because [organization redacted] is selling blood and injecting my child with a new medicine*.” (Mother in trial, FGD, Siaya)

Despite acknowledging the erroneousness of these objections, mothers who ultimately withdrew from the trial before their children received any vaccine described their limited capacity to confront misinformation in the household, ultimately conceding to their relatives’ demands to avoid further conflict.

*“I could not force because when the partner you are staying with does not approve [of] something, I cannot do it against his will. Although I was interested to enroll my child in the study, my husband and my mother in*-*law did not want [to]…Since they refused, and the child is also theirs, I could not force things.”* (Mother who withdrew, FGD, Wagai)

#### Information gaps surrounding the trial’s experimental design

Some mothers and fathers discussed the experimental nature of the trial as discouraging participation. A few participants, like this father, demonstrated confusion over the trial’s experimental design, which might have engendered skepticism over PfSPZ Vaccine:

*“Why couldn’t they give the same to government hospitals so that everyone gets it? Again, why did they select just a few children if it’s a vaccine for all children?”* (Father who withdrew, IDI, Wagai)

Similar to the blood draws, objections to trial participation were largely derived from fathers and in-laws who were not sensitized prior to mothers enrolling their children. As this mother explains, community members may have been confused by existing immunization standards, specifically their children’s experiences receiving other routine, non-experimental vaccines in government health facilities.

*“Some people talk negatively about malaria vaccine in the community…asking why healthy children were selected and not sick ones, while [the] government vaccinates all children in the hospital, since they all need vaccine.”* (Mother in trial, FGD, Siaya)

### DVI perceptions and experiences

#### Efficacy and tolerability

Many mothers whose children received PfSPZ Vaccine during the trial spoke favourably of the administration method, citing the vaccine’s tolerability relative to other intramuscularly administered childhood immunizations. Compared to other vaccines, many mothers reported that their children experienced less pain and rarely developed side effects post-inoculation.

*“When I took my child for vaccination at nine months in the government clinic, he did not sleep well and experienced pain…I thought that would be the same case with [the] malaria vaccine, but it was not the case. He played around and was just okay.”* (Mother in trial, FGD, Siaya)

*“Vaccines given through the vein are less painful as compared to the government methods. When they [IV vaccinations] are given to the child, sometimes the child develops fever…It is painful but not very painful. The child only cries during the injection.”* (Mother in trial, IDI, Siaya)

Of note, among interviewed caregivers, no child who received any dose of PfSPZ Vaccine was withdrawn from the trial because of the vaccination method. While mothers emphasized their children’s positive immunization experiences when discussing their preferences for DVI, study clinicians framed their support of DVI in terms of the method’s safety and efficacy.

*“The intravenous route is…is the best…Most drugs…are given orally, but when a drug is given intravenously…it is very effective because it goes directly into the system without still being absorbed in the gut…I think generally intravenous [administration] is as safe as any other route.”* (Clinician, IDI, Wagai)

#### Pain and unsuccessful vaccination attempts

PfSPZ Vaccine was generally accepted among participants whose children received it; however, there were concerns surrounding initial events of unsuccessful vaccine administration by DVI, which reduced over time with the improved clinician performance of the DVI procedure.

*“When they are looking for the vein, the child is pricked several times, and the child cries. As a mother, you sympathize, and there is nothing you can do…The child was looking at me with pain, but I was helpless…They should find a way of getting [the] vein without any harm to a child.”* (Mother in trial, IDI, Wagai)

However, these experiences were insufficient in motivating withdrawal from the trial. Apart from the pain elicited from unsuccessful vaccine administration attempts, unfavourable perceptions of and attitudes towards DVI were raised exclusively by mothers and fathers who had withdrawn the children from the trial prior to any vaccine being administered. These concerns mirrored objections of these participants to the blood draw (e.g., the procedure was too invasive or inappropriate for healthy children).

*“I have heard some say that the venous injection is meant for very sick children; therefore, it should be limited to them.”* (Village elder, IDI, Wagai)

#### Personnel and institutional constraints

Although they expressed support for DVI, study clinicians alluded to capacity and systemic challenges to rolling out PfSPZ Vaccine in the public sector. These included training requirements (to ensure administration success) and higher staffing requirements for administering the vaccine (one to restrain child, one to use the vein monitor, one to inject).

*“You can’t give it alone. You’ll need an assistant to hold the child for you…to hold the vein viewer [and] to give you…the vaccine…You must have…extra manpower unlike any [of] those other vaccines. You are just there with the mother…it’s also time consuming anyway compared to others because you have to look for the vein, [and] you have to keep the child…in a firm position.”* (Clinician, IDI, Wagai)

### Experiences with non-inoculation trial procedures

#### Care quality

Majority of mothers whose infants completed the three-dose inoculation series discussed the availability and quality of care provided as a major favourable element of the trial.

*“The treatment of the children is good such that when the doctors [from government hospitals] were on strike, I used to take my child to the hospital. Many people asked me where I was going while the doctors are on strike, and I told them that we have our own doctors there [study clinics].”* (Mother in trial, FGD, Wagai)

*“I like this study because if you bring a child, he’ll undergo all tests, rather than at other places. They’ll get the weight, height, temperature, and blood pressure…That made me happy knowing that even if I go there and go back, I know the state of my child.”* (Mother in trial, FGD, Wagai)

#### Provider and staff negative attitudes

Across FGDs with mothers whose children were enrolled in the trial, negative attitudes of study staff emerged as a salient theme, though this issue was limited to FGDs and not IDIs.

*“There was a day that I brought my child because she was vomiting at night. [After] reaching the hospital in the morning, she was okay. When I explained this to the doctor, there were some doctors saying that we just love money*–*that some women bring their children when not sick because they just wanted money. That really did hurt me.”* (Mother in trial, FGD, Wagai)

*“I have never liked how some doctors treat us in the clinic. Some really despise us…Some of them think that they are doing us favour. [They think] we can’t afford to treat our children and that we are so desperate for the study, yet they are the ones who came to us in the community.”* (Mother in trial, FGD, Siaya)

Participants who withdrew their children from the trial did not report any misunderstandings with or negative attitudes of study staff, suggesting this phenomenon was experienced exclusively by mothers with sustained contact with study clinicians.

#### Delays in trial procedures

Some, though fewer than half of, mothers of enrolled children reported frustrations with delays in trial procedures, including spending extensive hours at the clinic to have trial procedures administered.

*“I have never liked the…time we spend in the clinic. When you come late, they [the staff] quarrel. When you come early, no one bothers to attend to the child. Doctors just lock themselves in the room telling stories.*” (Mother in trial, FGD, Siaya)

Most caregivers, however, did not withdraw from the trial due to these perceived delays in trial procedures.

## Discussion

Qualitative insights gleaned from focus groups and interviews conducted with 112 caregivers, community members, and study clinicians in Siaya County, western Kenya reveal co-occurring enablers of and barriers to trial enrollment and sustained participation. Due to its tolerability, PfSPZ Vaccine’s DVI method was widely accepted by caregivers participating in the trial. Factors influencing early consent withdrawal from the trial were largely external to the DVI procedure and embedded in other aspects of the trial’s administration, most notably the protocol-required blood draws.

In this malaria-endemic setting, caregiver motivations to enroll their children in the experimental PfSPZ Vaccine trial revolved principally around safeguarding child health and averting financial hardships incurred from recurrent malaria infection. Studies from other high-prevalence settings highlight similar motivations underpinning acceptability and uptake patterns for malaria prevention commodities [30, 31]. Compared to other malaria prevention approaches that provide only intermittent protection or require sustained behaviour modification (e.g., IRS, LLINs) [30, 32], vaccines are generally perceived to provide prolonged immunity after a few doses [33, 34] and do not require protracted behaviour modification or routine engagement with health services. For these reasons, vaccines may appeal more strongly to caregivers than widely available malaria prevention strategies.

The blood draws emerged as a dominant barrier to trial participation and was the most frequently cited reason for pre-vaccination consent withdrawal. Objections to the blood draw stemmed almost exclusively from fathers and mothers-in-law, were founded in concerns surrounding infant safety, and were influenced by rumours circulating in the study communities surrounding malicious ulterior motives by study staff (e.g., selling children’s blood for money)—factors consistent with community objections to vaccines and experimental commodities documented in other trial/implementation settings [35–41]. Sustained parental and community anxieties around the blood draws reinforced skepticism and resulted in early participant termination. For context, in the parent PfSPZ Vaccine trial, 106 consent withdrawals occurred after initial consenting but prior to screening, where the first blood draw occurred, and one consent withdrawal occurred after screening but prior to vaccination. A total of 14 consent withdrawals occurred after vaccination. Importantly, the quantity of blood to be withdrawn at each study visit elevated the perceived risk of the procedure among mothers and fathers who withdrew their children.

The salience of parental objections to blood draws, coupled with the perceived preferential effectiveness of DVI compared to IM administration of PfSPZ Vaccine, highlights the centrality of systemic circulatory representations in decision-making surrounding trial participation and future vaccine uptake. A growing body of commentaries, ethnographies, and qualitative studies have interrogated trial non-participation and ethical considerations for collecting biological specimens in clinical research contexts. In the context of malaria vaccination, studies in Kenya [16] and Tanzania [42] reported the perceived *quantity* of blood to be drawn from study participants underpinned community reticence and, ultimately, fueled opposition to trial participation. Further exploring the role of “blood narratives” in trial non-participation, a qualitative study in rural Gambia explained how refusals to submit dried blood spots were rooted in concerns surrounding diminished corporeal strength, heightening perceived vulnerability to illness and poor health [41]. Objections to blood draws in the parent PfSPZ Vaccine trial, as documented extensively in other trial contexts, were importantly accompanied by rumours of unlicensed blood sharing and selling by trialists. Evidence from Gambia [41], Ghana [36], and Tanzania [42] suggest that while these rumours are oftentimes perpetuated by peripheral actors (i.e., marginalized individuals in communities unreached by available health services), the presence of these rumours are demonstrative of “social distancing” [43], or historically rooted asymmetries between health institutions and the communities in which they work. While this study could neither establish the origin of these rumours nor verify whether those perpetuating rumours participated in any trial mobilization or sensitization activities, the presence of these rumours, in spite of intentional efforts by study staff to communicate the purpose of these blood draws through trusted entities (i.e., community leaders, advisory boards), reveals unresolved sources of community mistrust in health institutions.

As study staff initially recruited mothers and requested consenting caregivers to sensitize their household members, future strategies to mitigate early withdrawal from trial participation should ensure heads of household and other family members with decision-making authority over child health are sensitized earlier and by the study staff directly. Despite continuous community sensitization during the trial to address emerging community rumours and concerns about the trial, these findings highlight the importance of carefully addressing these perceptions prior to enrollment and prioritizing individuals with decision-making authority in their household, not just the child’s primary caregiver, in sensitization activities [29, 31, 34]. Investing in community-led mobilization activities highlighting the safety and appropriateness of the blood draws can help validate salient community concerns and rebuild trust in research processes [30, 33, 35].

Among participants whose infants received any dose of vaccine or placebo intravenously, the DVI method was widely accepted across participant subgroups with no apparent differences in acceptability by socio-demographic categories or study site. Compared to IM vaccines, the DVI method was favoured for its high tolerability during and after infant inoculation. Nearly all caregiver objections to DVI stemmed from child reactions to repeated vaccination attempts, which occurred initially but were mitigated over time with improved clinical expertise. In light of observed sensitivities to blood draws in the trial context, caregiver preferences for DVI is a meaningful finding, as this administration route can improve the marketability and amenability of novel infant immunizations, like PfSPZ Vaccine, in a context where this inoculation method has properties deemed more effective and tolerable than traditional IM administration. Clinicians, likewise, appreciated caregiver acceptance of DVI administration of the vaccine; however, they expressed concerns over the feasibility of scaling PfSPZ Vaccine nationally in a standard EPI-like immunization programme due to personnel constraints and administration requirements.

Sources of caregiver dissatisfaction with the trial among those who continued participating highlight potential issues to consider in the design of future trials and programmes. Mothers whose children were enrolled in the trial reported negative attitudes by study staff as a source of dissatisfaction with the trial. Delays in administering trial procedures similarly dissuaded and, only in a handful of cases, motivated caregivers to terminate participation. While no caregivers reported withdrawing a child from the trial as a result of perceived misunderstandings with clinicians, these experiences, nonetheless, highlight needs for participant sensitivity training with staff, managerial oversight of interactions with participants, and improved client-flow during vaccine visits.

This study fills substantial gaps in the literature on barriers and facilitators to enrollment and participation in a trial of a novel malaria vaccine with a non-traditional route of administration (i.e., venous inoculation). There is a dearth of literature documenting caregiver/community experiences with malaria vaccination, particularly in trial contexts. A majority of the malaria vaccine acceptability literature formatively assesses perceptions and preferences for prospective immunization modalities and delivery strategies [16–21, 33, 44, 45]. While findings are essential to identifying user-amenable malaria prevention commodities and delivery strategies, these aforementioned studies cannot elucidate lived community experiences with a candidate malaria vaccine in research or programmatic settings. This study is among the first to provide insights into caregiver, community, and clinician experiences with the PfSPZ Vaccine, whose atypical DVI administration route raises new considerations in the development and deployment of community sensitization and mobilization strategies.

### Limitations

Results from this qualitative study must be appraised in light of several limitations. Regarding participant sampling, a number of eligible clinicians were unavailable at the time of recruitment or refused to participate in the study, yielding a smaller clinician sample (n = 3) compared to other participant subgroups. This compromises the ability to gauge whether data saturation was reached for this subgroup. Additionally, due to time and resource constraints, investigators could not recruit other subgroups, specifically mothers-in-law, with capacity to influence decision-making surrounding trial participation and receipt of PfSPZ Vaccine. Inclusion of these perspectives may have supplemented current findings with added nuances into trial perceptions influencing acceptance or refusal of trial procedures. Furthermore, the timing of data collection (close to trial close-out) may have amplified recall bias, as participants shared perspectives on events occurring as early as 1 year prior to data collection. The timing of study initiation and recruitment also restricted capacity to leverage other robust qualitative data collection methods, namely direct observation of consent procedures and infant vaccination, to deepen insights and triangulate findings from IDIs and FGDs. Lastly, while an overarching objective of achieving data richness (thematic depth over breadth) informs sampling and data collection procedures in qualitative studies like this one, these results do not necessarily capture the full panorama of potential perspectives and experiences with trial recruitment procedures, participation, or PfSPZ Vaccine administration by DVI.

## Conclusions

This study highlights important enablers of and barriers to PfSPZ Vaccine uptake and sustained trial engagement, providing meaningful insights into the acceptability and feasibility of PfSPZ Vaccine and its accompanying trial procedures. There is high caregiver acceptability of PfSPZ Vaccine’s DVI method, despite perceived pain by caregivers in some vaccination episodes. The findings provide meaningful insights into strategies to improve trial participation and participant retention by explaining the necessity of trials and more clearly illustrating and discussing testing involved (e.g., the volume of blood removed during the trial is not harmful to small children). Community concerns and objections to the trial procedures (e.g., blood draw), specifically from non-sensitized household and community members, emphasize the need to integrate these populations into mobilization and sensitization strategies to enhance community trust and increase trial acceptability. Participant (dis)satisfaction should also be monitored at the beginning of future trials to more expediently rectify issues and prevent additional withdrawals.

## Data Availability

The datasets used and/or analysed during the current study are available from the corresponding author upon reasonable request.

## Abbreviations

ACT: Artemisinin-based combination therapy
DVI: Direct venous inoculation
IM: Intramuscular
IRS: Indoor residual spraying
LLIN: Long-lasting insecticidal net
PfSPZ: *Plasmodium falciparum* sporozoite

## References

[1] WHO. World Malaria Report, 2018 [Internet]. Geneva, World Health Organization; 2018 [cited 2017 Nov 21]. Available from: https://apps.who.int/iris/bitstream/handle/10665/275867/9789241565653-eng.pdf?ua=1.

[2] Lengeler C. Insecticide-treated bed nets and curtains for preventing malaria. Cochrane Database Syst Rev. 2004;2:CD000363. 10.1002/14651858.CD000363.pub2 15106149

[3] Pluess B, Tanser FC, Lengeler C, Sharp BL. Indoor residual spraying for preventing malaria. Cochrane Database Syst Rev. 2010;4:CD006657. 10.1002/14651858.CD006657.pub2 PMC6532743 20393950

[4] Sinclair D, Zani B, Donegan S, Olliaro P, Garner P. Artemisinin-based combination therapy for treating uncomplicated malaria. Cochrane Database Syst Rev. 2009;3:CD007483. 10.1002/14651858.CD007483.pub2 PMC6532584 19588433

[5] Aponte JJ, Schellenberg D, Egan A, Breckenridge A, Carneiro I, Critchley J (2009). Efficacy and safety of intermittent preventive treatment with sulfadoxine-pyrimethamine for malaria in African infants: a pooled analysis of six randomised, placebo-controlled trials. Lancet.

[6] Greenwood B (2006). Intermittent preventive treatment–a new approach to the prevention of malaria in children in areas with seasonal malaria transmission. Trop Med Int Health..

[7] ter Kuile FO, van Eijk AM, Filler SJ (2007). Effect of sulfadoxine-pyrimethamine resistance on the efficacy of intermittent preventive therapy for malaria control during pregnancy: a systematic review. JAMA.

[8] WHO. Global Technical Strategy for Malaria 2016–2030 [Internet]. Geneva, World Health Organization; 2015 Jun [cited 2017 Nov 21]. Available from: http://apps.who.int/iris/bitstream/10665/176712/1/9789241564991_eng.pdf?ua=1&ua=1.

[9] Hoffman SL, Billingsley PF, James E, Richman A, Loyevsky M, Li T (2010). Development of a metabolically active, non-replicating sporozoite vaccine to prevent *Plasmodium falciparum* malaria. Hum Vaccin..

[10] Epstein JE, Tewari K, Lyke KE, Sim BKL, Billingsley PF, Laurens MB (2011). Live attenuated malaria vaccine designed to protect through hepatic CD8^+^ T cell immunity. Science.

[11] Seder RA, Chang L-J, Enama ME, Zephir KL, Sarwar UN, Gordon IJ (2013). Protection against malaria by intravenous immunization with a nonreplicating sporozoite vaccine. Science.

[12] Sissoko MS, Healy SA, Katile A, Omaswa F, Zaidi I, Gabriel EE (2017). Safety and efficacy of PfSPZ Vaccine against *Plasmodium falciparum* via direct venous inoculation in healthy malaria-exposed adults in Mali: a randomised, double-blind phase 1 trial. Lancet Infect Dis..

[13] Jongo SA, Shekalaghe SA, Church LWP, Ruben AJ, Schindler T, Zenklusen I (2018). Safety, immunogenicity, and protective efficacy against controlled human malaria infection of *Plasmodium falciparum* sporozoite vaccine in Tanzanian Adults. Am J Trop Med Hyg.

[14] Dimala CA, Kika BT, Kadia BM, Blencowe H (2018). Current challenges and proposed solutions to the effective implementation of the RTS, S/AS01 malaria vaccine program in sub-Saharan Africa: a systematic review. PLoS ONE.

[15] White SE, Harvey SA, Meza G, Llanos A, Guzman M, Gamboa D (2018). Acceptability of a herd immunity-focused, transmission-blocking malaria vaccine in malaria-endemic communities in the Peruvian Amazon: an exploratory study. Malar J..

[16] Ojakaa DI, Ofware P, Machira YW, Yamo E, Collymore Y, Ba-Nguz A (2011). Community perceptions of malaria and vaccines in the South Coast and Busia regions. Malar J..

[17] Bingham A, Gaspar F, Lancaster K, Conjera J, Collymore Y, Ba-Nguz A (2012). Community perceptions of malaria and vaccines in two districts of Mozambique. Malar J..

[18] Chukwuocha UM, Okorie PC, Iwuoha GN, Ibe SN, Dozie IN, Nwoke BE (2018). Awareness, perceptions and intent to comply with the prospective malaria vaccine in parts of South Eastern Nigeria. Malar J..

[19] Mtenga S, Kimweri A, Romore I, Ali A, Exavery A, Sicuri E (2016). Stakeholders’ opinions and questions regarding the anticipated malaria vaccine in Tanzania. Malar J..

[20] Meñaca A, Tagbor H, Adjei R, Bart-Plange C, Collymore Y, Ba-Nguz A (2014). Factors likely to affect community acceptance of a malaria vaccine in two districts of Ghana: a qualitative study. PLoS ONE.

[21] Kpanake L, Sorum PC, Mullet E (2016). The potential acceptability of infant vaccination against malaria: a mapping of parental positions in Togo. Vaccine..

[22] National Malaria Control Programme, Ministry of Health, Kenya National Bureau of Statistics. Kenya Malaria Indicator Survey (MIS), 2015. [Internet]. Nairobi, 2016. Available from: https://dhsprogram.com/pubs/pdf/MIS22/MIS22.pdf.

[23] RTS,S Clinical Trials Partnership. Efficacy and safety of RTS,S/AS01 malaria vaccine with or without a booster dose in infants and children in Africa: final results of a phase 3, individually randomised, controlled trial. Lancet. 2015;386:31–45. 10.1016/S0140-6736(15)60721-8 PMC5626001 25913272

[24] Otieno L, Oneko M, Otieno W, Abuodha J, Owino E, Odero C (2016). Safety and immunogenicity of RTS, S/AS01 malaria vaccine in infants and children with WHO stage 1 or 2 HIV disease: a randomised, double-blind, controlled trial. Lancet Infect Dis..

[25] Crabtree BF, Miller WL. Doing Qualitative Research. SAGE Publications; 1999. 432 p.

[26] Gale NK, Heath G, Cameron E, Rashid S, Redwood S (2013). Using the framework method for the analysis of qualitative data in multi-disciplinary health research. BMC Med Res Methodol.

[27] Ayres L, Kavanaugh K, Knafl KA (2003). Within-case and across-case approaches to qualitative data analysis. Qual Health Res.

[28] Corbin J, Strauss A (2008). Basics of Qualitative Research: Techniques and Procedures for Developing Grounded Theory.

[29] Miles MB, Huberman AM, Saldaña J (2014). Qualitative Data Analysis: A Methods Sourcebook.

[30] Toé LP, Skovmand O, Dabiré KR, Diabaté A, Diallo Y, Guiguemdé TR (2009). Decreased motivation in the use of insecticide-treated nets in a malaria endemic area in Burkina Faso. Malar J..

[31] Watiro AH, Awoke W (2016). Insecticide-treated net ownership and utilization and factors that influence their use in Itang, Gambella region, Ethiopia: cross-sectional study. Risk Manag Healthc Policy..

[32] Magaço A, Botão C, Nhassengo P, Saide M, Ubisse A, Chicumbe S (2019). Community knowledge and acceptance of indoor residual spraying for malaria prevention in Mozambique: a qualitative study. Malar J..

[33] Febir LG, Asante KP, Dzorgbo D-BS, Senah KA, Letsa TS, Owusu-Agyei S. Community perceptions of a malaria vaccine in the Kintampo districts of Ghana. Malar J. 2013;12:156. 10.1186/1475-2875-12-156 PMC3656774 23651533

[34] Kagoné M, Yé M, Nébié E, Sié A, Müller O, Beiersmann C (2018). Community perception regarding childhood vaccinations and its implications for effectiveness: a qualitative study in rural Burkina Faso. BMC Public Health..

[35] Boahen O, Owusu-Agyei S, Febir LG, Tawiah C, Tawiah T, Afari S (2013). Community perception and beliefs about blood draw for clinical research in Ghana. Trans R Soc Trop Med Hyg.

[36] Chatio S, Baiden F, Achana FS, Oduro A, Akazili J (2016). Knowledge and perceptions about clinical trials and the use of biomedical samples: findings from a qualitative study in rural Northern Ghana. PLoS ONE.

[37] Compaoré A, Dierickx S, Jaiteh F, Nahum A, Bohissou TFE, Tinto H (2018). Fear and rumours regarding placental biopsies in a malaria-in-pregnancy trial in Benin. Malar J..

[38] Fairhead J, Leach M, Small M (2006). Public engagement with science? Local understandings of a vaccine trial in the Gambia. J Biosoc Sci.

[39] Kingori P, Muchimba M, Sikateyo B, Amadi B, Kelly P (2010). “Rumours” and clinical trials: a retrospective examination of a paediatric malnutrition study in Zambia, southern Africa. BMC Public Health..

[40] Newton S, Doku V, Geissler W, Asante KP, Cousens S (2009). Drawing blood from young children: lessons learned from a trial in Ghana. Trans R Soc Trop Med Hyg.

[41] O’Neill S, Dierickx S, Okebe J, Dabira E, Gryseels C, d’Alessandro U (2016). The importance of blood is infinite: conceptions of blood as life force, rumours and fear of trial participation in a Fulani village in Rural Gambia. PLoS ONE.

[42] Liheluka EA, Lusingu JP, Manongi RN (2013). Community perceptions on the secondary health benefits established by malaria vaccine trials (RTS, S phase 2 and phase 3) at the Korogwe site in North Eastern Tanzania. Malar J..

[43] Peeters Grietens K, Ribera JM, Erhart A, Hoibak S, Ravinetto RM, Gryseels C (2014). Doctors and vampires in sub-Saharan Africa: ethical challenges in clinical trial research. Am J Trop Med Hyg.

[44] Ojakaa DI, Jarvis JD, Matilu MI, Thiam S (2014). Acceptance of a malaria vaccine by caregivers of sick children in Kenya. Malar J..

[45] Romore I, Ali AM, Semali I, Mshinda H, Tanner M, Abdulla S (2015). Assessment of parental perception of malaria vaccine in Tanzania. Malar J..

